# Central post-stroke pain: predictors and relationship with magnetic resonance imaging and somatosensory evoked potentials

**DOI:** 10.1186/s41983-018-0041-z

**Published:** 2018-12-03

**Authors:** Ahmed Osama, Ahmed Abo Hagar, Saly Elkholy, Mohamed Negm, Reda Abd El-Razek, Marwa Orabi

**Affiliations:** 1 Neurology, Faculty of Medicine, Suez Canal University, Ismailia, Egypt; 2 Clinical Neurophysiology, Cairo University, Cairo, Egypt

**Keywords:** Stroke, Central post-stroke pain, Short latency somatosensory evoked potentials

## Abstract

**Background:**

Central post-stroke pain (CPSP) is an under-recognized complication of stroke although it can lead to deterioration in quality of life and impairment in activities of daily living. Its estimated prevalence varies between 18.6 and 49%.

**Objective:**

To investigate the prevalence and predictors of CPSP in ischemic stroke patients and to find its relationship with somatosensory evoked potentials (SSEPs) and magnetic resonance imaging.

**Patients and methods:**

Sixty five consecutive patients with recent first attack of ischemic stroke who were admitted to the Neurology Department, Suez Canal University Hospitals were recruited. Patients were subjected to clinical assessment, Hamilton depression rating scale, brain MRI, short-form McGill Pain Questionnaire (SF-MPQ), daily pain rating scale (DPRS), stimulus evoked pain, and SSEPs.

**Results:**

The total prevalence rate of CPSP was 35.4% (*n* = 23). The mean age of the patients developed CPSP was significantly lower than those without CPSP (*p* = 0.004). Deep sensory dysfunction was statistically significantly higher among CPSP group than non-CPSP group (*p* = 0.001). CPSP group showed statistically significant higher prevalence of thalamic stroke (*p* = 0.007), as well as significant abnormalities in inter-peak interval (IPL) of median and tibial nerves SSEPs (*p* < 0.05). Thalamic group showed higher abnormalities in IPL of median and tibial nerves compared to extra-thalamic group, but without statistically differences.

**Conclusion:**

The prevalence of CPSP was found to be 35.4%. Predictors of CPSP include; deep sensory dysfunction, prolongation of tibial N21–P40 IPL, smoking history, age < 50 years, presence of thalamic stroke and prolongation of median N9–N20 IPL.

## Introduction

After stroke, central post-stroke pain (CPSP) is not an uncommon complication which may interfere with quality of life. Post-stroke pain (PSP) is a common but not take much attention in stroke research; in different studies its prevalence varies from 18.6 to 49% [1]. The criteria most commonly included in CPSP are “Development of pain with onset at or after the stroke, Pain located on the stroke-affected side of the body and No other plausible cause of the pain, including pain isolated to the shoulder joint and nearby region” [2]. CPSP may be produced by lesions at any part of the spinothalamocortical pathways (known before as thalamic pain syndrome). Most of patients develop CPSP within 6 months after stroke [3].

SSEPs could be of value in detection and assessment of CPSP. SSEPs may document a lesion in the central sensory and lemniscal pathways in patients with CPSP, and give information on the pathological process (demyelination, degenerative), the site of the lesion (spinal cord, brain), and the presence of subclinical involvement at other sites. They have diagnostic value, because most diseases causing CPSP do not result in damage or lesion limited to the nociceptive system, but extend to the whole peripheral nerve or involve non-nociceptive pathways [4].

The aim of the work is to investigate the prevalence of central post-stroke pain (CPSP) in ischemic stroke patients, its predictors, and its relationship with somatosensory evoked potentials (SSEPs) and magnetic resonance imaging.

## Patients and methods

The study included 65 consecutive patients with recent first attack of unilateral ischemic stroke admitted to the Neurology Department, Suez Canal University Hospital. We included both genders, age ≥ 18 years. We excluded patients with diabetes, history of old cerebrovascular stroke, disturbed level of consciousness, dementia, aphasia to an extent that patients could not explain themselves, degenerative neurological diseases, peripheral neuropathy, history of brain tumors, history of autoimmune disease, history of psychiatric disorders, and patients showing signs of peripheral neuropathy on nerve conduction studies.

All selected patients were evaluated within 7 days after stroke onset and followed at periodic intervals (1 month, 3 months, and 6 months after stroke). The following were done:

A- Clinical assessment:

1. The onset of CPSP from the day of stroke, distribution, character, and severity of pain
2. Sensory testing—sensory testing was done first on the normal side followed by abnormal sides. Pinprick and touch was tested. For temperature testing, cold metal rod, and for vibration, a 128-Hz tuning fork was used. Joint position sense was tested in toes and fingers using 1° deflection.
3. Hamilton depression rating scale (HAM-D scale), a multiple choice questionnaire was used to evaluate and to demonstrate the severity of depression [5].

### Diagnostic criteria for CPSP

CPSP criteria to be evaluated for each patient based on a grading system for neuropathic pain by Treede and co-workers (2008) [6] are as follows:

1. Other common causes of pain exclusion.
2. Pain with a special neuro-anatomically apparent distribution: Either pain confined in one side in the body and/or face or one on one side of the body with other side involvement of the face.
3. A history revealing stroke: Sudden occurrence of neurological symptoms with pain onset at or after stroke
4. Signs of the special neuro-anatomically apparent distribution by neurological examination: Prediction of sensory negative or positive signs in the painful area, unprompted and/or evoked pain localized within a branch of sensory abnormality, and anatomically apparent distribution of sensory dysfunction
5. Pointing to relevant vascular lesion by imaging: CT or MR brain shows a lesion that can explain the sensory findings

Possible CPSP is diagnosed if criteria 1, 2, and 3 are met. Probable CPSP is diagnosed if criteria 1, 2, and 3 plus either criteria 4 or 5 are met. Definite CPSP is diagnosed if criteria 1–5 are met.

B- MRI brain: Carried out by Philips Achieva 1.5 Tesla MRI scanner, Germany. It was done within 1 week of the onset of stroke. T2, T1, and diffusion weighted images in the sagittal and axial planes were obtained. Lesions detected by MRI were grouped into thalamic and extra-thalamic.
C- Quantitative assessment of neuropathic pain. We used the following scales:
  Short-form McGill Pain Questionnaire (SF-MPQ): The severity of CPSP was quantified by SF-MPQ**.** The SF-MPQ, a shorter version of the MPQ, is a multidimensional measure of perceived pain in adults with chronic pain. The SF-MPQ consists of three items: pain rating index (PRI), present pain intensity (PPI), and visual analog scale (VAS).
  Daily pain rating scale (DPRS) is 0–10 cm, in which 0 is absent of pain and 10 is the worst pain in last week. The patients recorded the DPRS every day in the last week before the following visit and average was calculated.
  Stimulus-evoked pain was evaluated for static mechanical allodynia, dynamic mechanical allodynia, cold allodynia, and punctate hyperalgesia.
D- Somatosensory evoked potential (SSEPs) studies were done using the Neuropack X1 EMG/EP measuring system MEB-2300, NIHON KOHDEN machine, 4 channels. They were performed for all patients on eligibility (baseline) then performed to the diagnosed patients with CPSP during follow-up and finally performed for the patients who do not experience CPSP at the end of follow-up (6 months).

1. Median SSEP was recorded from the affected hemiparetic limb. Median nerve was stimulated at wrist by 0.1-ms square-wave pulse at 3 Hz, the intensity which make the thumb twitch with no pain. At Erb’s point, active electrode for surface recording was placed, and at opposite parietal cortex 3 cm behind and 7 cm lateral to vertex, mid frontal (Fz) reference was used. For the evaluation of median SSEP, N9 and N20 peak latencies and N9–N20 IPL were recorded.
2. Tibial SSEPs was recorded from the affected hemiparetic limb. Posterior tibial nerve was stimulated under the medial malleolus by 0.1-ms square-wave pulse at 3 Hz, the intensity which make the great toe twitch with no pain. At the spinous process, the recording electrodes were placed on L1 vertebra and 2 cm caudal to Cz’. At L3 and Fz respectively, the reference electrode was placed. For the evaluation of tibial SEP, N21 and P40 peak latencies and N21–P40 IPL were recorded [7].

#### Ethics approval and consent to participate

Suez Canal Faculty of Medicine Ethical Committee approved this study in 26/11/2012; number of approval is 831, and all participants signed informed written consent before participating in the study.

#### Statistical analysis

Using SPSS IBM SPSS statistics, collected data were processed (version 22.0, 2013; IBM Corp. Armonk, NY, USA). Qualitative data were uttered as numbers and percentages and quantitative data were uttered as means ± SD. To test significance of difference between two means, unpaired *t* test was used; while to test significance of difference between qualitative data, chi-square was used. A probability value (*p* value) < 0.05 was considered statistically significant; while a probability value (*p* value) < 0.01 was considered statistically highly significant. Odds ratio (OR) and 95% confidence interval (CI) were assessed using ordinal regression analysis model to test the best fitting predictors.

## Results

**Fig. 1:**
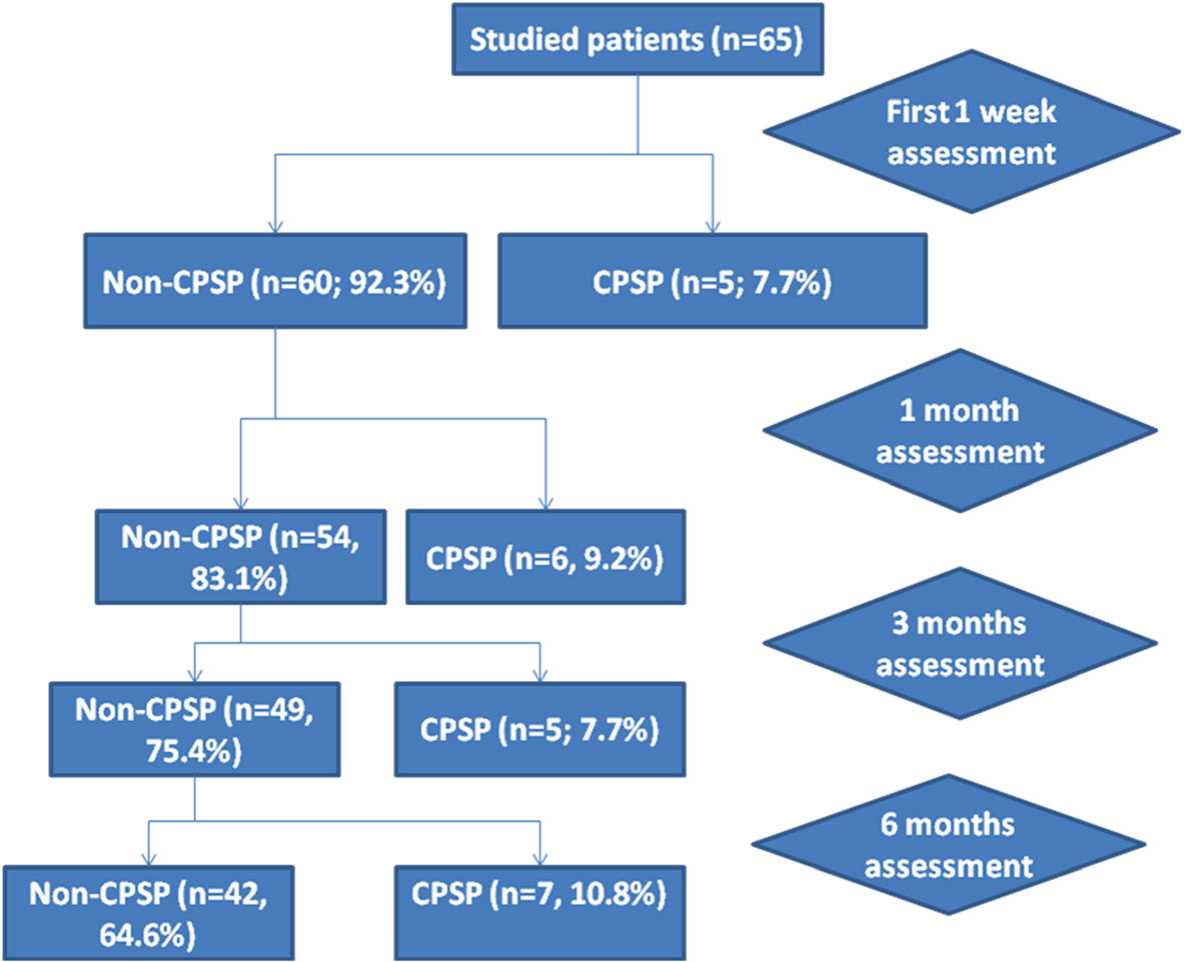
Incidence rate of central post-stroke pain (CPSP) during follow-up

The patients’ mean age was 59.6 ± 10.4 years. Male represented 64.6% while females were 35.4%. Though 15.4% of the patients were smokers and 53.8% was hypertensive. Twenty-three patients developed CPSP with prevalence rate of 35.4% that was subdivided as follows: 7.7% during the first week post-stroke, 9.2% during the first month, 7.7% during the third month, and 10.8% during the sixth month of follow-up (Fig. 1).

The mean age of the patients who developed CPSP (53.6 + 9.6) was statistically lower than those who did not (62.8 + 9.4, *p* value = 0.004), with no gender difference. Smokers developed CPSP more than non-smokers (30.4% versus 7.1%, *p* = 0.026). Hypertension, AF, and TIA were not discovered to be significantly different in both groups (*p* > 0.05). Meanwhile, the prevalence of ischemic heart disease was significantly higher among patients without CPSP (*p* = 0.043). There were no statistically significant differences between both groups regarding depression (30.4% versus 28.6%, *p* > 0.05).

Motor dysfunction, superficial, and cortical sensory loss showed no significant differences between both groups. But deep sensory dysfunction was statistically higher among patients with CPSP than patients without (*p* = 0.001) (Table 1).

**Table 1:** Clinical characteristics and location of stroke according to (MRI)

Variables	Patients with CPSP (*n* = 23)	Patients without CPSP (*n* = 42)	Test used	*p* value
	No. (%)	No. (%)		
Motor dysfunction	15 (65.2%)	25 (59.5%)	*χ*^*2*^ = 0.20	0.65
Superficial sensory loss	11 (47.8%)	17 (40.5%)	*χ*^*2*^ = 0.33	0.56
Deep sensory loss	6 (26.1%)	0 (0.0%)	Fisher	0.001**
Cortical sensory loss	2 (8.7%)	0 (0.0%)	Fisher	0.12
Location of lesion				
Thalamic	11 (47.8%)	7 (16.7%)	*χ*^*2*^ = 7.2	0.007**
Extra-thalamic	12 (52.2%)	35 (83.3%)		
Cortical	2 (8.7%)	8 (19.0%)	Fisher	0.31
Sub-cortical	3 (13.0%)	9 (21.4%)	Fisher	0.52
Capsular	2 (8.7%)	8 (19.0%)	Fisher	0.31
Basal ganglion	2 (8.7%)	5 (11.9%)	Fisher	0.52
Pontine	2 (8.7%)	3 (7.1%)	Fisher	0.59
Medullary	1 (4.3%)	2 (4.8%)	Fisher	0.72
Side of lesion				
Right side	11 (47.8%)	27 (64.3%)	*χ*^*2*^ = 1.7	0.20
Left side	12 (52.2%)	15 (35.7%)		

**Highly significant *p* value at < 0.01, *CPSP* central post-stroke pain, Fisher exact probability test, *χ*^*2*^ chi-square test

Patients with CPSP showed statistically significant higher prevalence of thalamic stroke (47.8%) (*p* = 0.007) (Table 1).

The left-sided lesion was significantly more in patients with thalamic compared to extra-thalamic affection (72.7% versus 33.3%, respectively) (Table 2).

**Table 2:** (MRI) findings according to side of lesion and site of pain

Variables	Thalamic (*n* = 11)	Extra-thalamic (*n* = 12)	Used test	*p* value
	No. (%)	No. (%)		
Side of lesion				
Right side	3 (27.3%)	8 (66.7%)	*χ*^*2*^ = 4.0	0.048*
Left side	8 (72.7%)	4 (33.3%)		
Site of pain				
Hemi-body including face	2 (18.2%)	6 (50.0%)	Fisher	0.048*
Hemi-body not including face	7 (63.6%)	3 (25.0%)	Fisher	0.039*
Lower limbs	2 (18.2%)	1 (8.3%)	Fisher	0.59
Upper limbs	0 (0.0%)	2 (16.7%)	Fisher	0.48

*Significant *p* value at < 0.05, *Fisher* Fisher exact probability test, *χ*^*2*^ chi-square test

According to SSEP; the mean peak latency and IPL differences were prolonged in patients with CPSP with highly statistically significant difference between both groups (*p* < 0.01) (Table 3).

**Table 3:** SSEP; of median and tibial nerves

Variables	Patients with CPSP (*n* = 23)	Patients without CPSP (*n* = 42)	*t* test	*p* value
Median N9				
Mean ± SD	11.0 ± 1.6	9.9 ± 1.1	3.3	0.002**
Range	8–14	8–12		
Median N20				
Mean ± SD	22.4 ± 2.8	20.1 ± 1.1	4.7	< 0.0001**
Range	18–26	19–23		
Median N9–N20 IPL				
Mean ± SD	11.5 ± 1.6	10.2 ± 0.93	4.2	0.0001**
Range	9–14	8–12		
Tibial N21				
Mean ± SD	22.8 ± 2.0	20.5 ± 0.99	7.1	< 0.0001**
Range	20.3–26.7	18–22		
Tibial P40				
Mean ± SD	44.4 ± 3.3	40.8 ± 1.1	4.5	< 0.0001**
Range	41.1–51.4	40–51		
Tibial N21–P40 IPL				
Mean ± SD	21.6 ± 1.9	20.3 ± 0.11	4.4	< 0.0001**
Range	17.4–25.5	19–21		

**Significant *p* value at < 0.01, *CPSP* central post-stroke pain, *SSEP* somatosensory evoked potential, *SD* standard deviation, *t* Student’s (paired) *t* test

Thalamic location group showed higher abnormalities in SSEP-IPL of median and tibial nerves compared to extra-thalamic group, but without statistically significant differences.

The best fitting factors significantly predict CPSP and include presence of deep sensory dysfunction (OR 14.5), tibial N21–P40 IPL > 21 ms (OR 8.4), smoking history (OR 5.7), age < 50 years (OR 5.1), presence of thalamic stroke (OR 4.6), and median N9–N20 IPL > 10.8 ms (OR 3.9) (Table 4).

**Table 4:** Predictors of central post-stroke pain (CPSP)

Variables	OR	95% CI	*p* value
Tibial N21–P40 IPL > 21.0 ms	8.4	2.0–35.4	0.003**
Deep sensory dysfunction	14.5	1.6–129.5	0.006**
Thalamic stroke	4.6	1.5–14.5	0.007**
Median N9–N20 IPL > 10.8 ms	3.9	1.3–12	0.015*
Age < 50 years	5.1	1.3–19.4	0.019*
Smoking history	5.7	1.3–24.8	0.026*

*Significant *p* value at < 0.05, **significant *p* value at < 0.01, *IPL* inter-peak latencies, *CPSP* central post-stroke pain, *OR* odds ratio, *CI* confident intervals

## Discussion

CPSP remains to be an under-recognized sequel of stroke although it can lead to deterioration in quality of life and impairment in activities of daily living [8]. In this study, the CPSP prevalence rate was 35.4% that was subdivided as follows: 7.7% during the first week post-stroke, 9.2% during the first month, 7.7% during the third month, and 10.8% during the sixth month of follow-up. Prevalence of CPSP is reported between 8% and 35% [9] so our results are nearly within the rated prevalence of CPSP. Other studies reported that after stroke, time to CPSP onset varies considerably. Hansson, 2004 [10] and Leijon et al. 1989 [11] mentioned that CPSP onset was “immediate in 15% (4/27) of patients, occurred within the first month in 37%, and between 1 and 34 months in the remaining 48%; in 78% of cases, CPSP onset occurred within 3 months.” Andersen et al. 1995 [12] pointed that “CPSP onset occurred within 1 month in 63% (10/16) of patients, between 1 and 6 months in 19% (3/16), and at more than 6 months in 19% (3/16)”. Similarly, Nasreddine and Saver 1997 [13] reported that “CPSP initiated within the first week in 36%, at 1 week to 1 month in 20%, and at 1-6 months in 27%.” Although CPSP occurs predominantly in the first 6 months, it can occur up to 10 years after stroke [14]. Seifert et al. 2013 [15] mentioned that CPSP can persist for many years or even throughout life.

In our study, the mean age of the patients who developed CPSP was 53.6 + 9.6 compared to 62.8 + 9.4 on other group; some studies indicate that CPSP is more prevalent in younger patients [2, 3, 8, 11] and other studies considered that the prevalence of CPSP is not related to gender, age, or side of lesion [12].

The younger age of development of CPSP can be explained by that posterior territory infarcts, including brainstem and thalamic strokes, which are frequently associated with CPSP are relatively more common in the young age [3, 9]. Also, sensitivity to heat pain is higher in young age, whereas in the elderly, sensitivity to pressure pain was augmented [16]: finally, young age had lower pain threshold due to faster conduction so with increasing age, the thresholds of non-noxious stimuli increase [1, 17].

In this study, no gender difference in both groups was detected, same results reported by Andersen et al. 1995 [12], on the other hand, gender was identified as predictor in other studies [2, 8]. In this study, no significant association between depression and CPSP was detected; the same finding was reported by Andersen and colleague1995 [12], Mukherjee and colleague 1999 [18], and Naess and colleague 2010 [16]. While Heutink and colleague 2010 [19] reported that like pains and medical conditions, the CPSP experience may be affected by psychosocial factors.

In our study, smoking was a trigging factor, same results reported by Misra and colleague [17]. Deep sensory loss was the significant clinical predictor for the development of CPSP; the same finding was reported by Meschia and Bruno (1998) [20]. Although statistically non-significant the percentage of motor dysfunction was similar to that detected by previous studies [17, 21–23].

In this study, 47.8% of CPSP patients has thalamic location and 52.2% extrathalamic; the same finding was reported by Boivie et al. 1989 [24] who demonstrated that this type of pain has a complex pathophysiology. Thus, while the thalamus is still recognized as key pathophysiological component, radiological techniques have shown lesions which have led to CPSP may be located at any level along the neuraxis.

Central disinhibition, especially at the thalamic level, can cause CPSP. Activity of medial thalamus could be inhibited by lesions of lateral thalamus and cause pain via disturbance of inhibitory pathways between medial and lateral pathways. Inhibitory inter-neurons in thalamic reticular nuclei explain an indirect route of such disinhibition [25].

According to SSEP, our study demonstrates that the mean peak latency and IPL differences were prolonged in patients with CPSP with highly statistically significant difference between both groups. Other studies mentioned that SSEPs are unaffected in Wallenberg’s syndrome and in some of the hemispheric but are generally abnormal when medial lemniscus is involved [26]. On the other hand, Kumar et al. (2009) [9] demonstrated that SSEP shows complete loss of contralateral cortical response but maintenance of P9, P14, and N18 far-fields in one third of their CPSP patients. Also, Misra et al. 2008 [17] reported that SSEPs were abnormal in 68% of CPSP; higher rate of SSEP abnormality in their study may be explained by that they take both hemorrhagic and ischemic stroke.

Using regression analysis, predictors of CPSP were best fit to the presence of deep sensory dysfunction, smoking history, age < 50 years, thalamic stroke, prolonged tibial N21–P40 IPL, and prolonged median N9–N20 IPL. Similar results reported by Klit et al. 2014 [27] who find that early evoked pain (dysesthesia)—“an unpleasant abnormal sensation produced by normal stimuli”—is a predictor for CPSP. Misra and colleague [17] reported that smoking was a trigging factor for CPSP. Many studies indicate that CPSP is more prevalent in younger patients [2, 3, 8, 11]. Boivie et al. 1989 [24] reported that CPSP patients has been reported in thalamic location than in other locations. Misra et al. 2008 [17] reported that SSEPs were abnormal in 68% of CPSP.

Limitations of the study includes that large number of patients can be included, longer time of follow-up, management strategy and drugs used for every patient, and social factors for every patient.

## In conclusion

The prevalence rate of CPSP was 35.4%. Predictors of CPSP include deep sensory dysfunction, prolongation of tibial N21–P40 IPL, smoking history, age < 50 years, presence of thalamic stroke, and prolongation of median N9–N20 IPL.

## Abbreviations

CPSP: Central post-stroke pain
DPRS: Daily pain rating scale
HAM-D scale: Hamilton depression rating scale
MRI: Magnetic resonance imaging
PSP: Post-stroke pain
SF-MPQ: Short-form McGill Pain Questionnaire
SSEPs: Somatosensory evoked potentials
